# Fluconazole induced toxic epidermal necrolysis: a case report

**DOI:** 10.1186/1757-1626-2-9071

**Published:** 2009-11-20

**Authors:** Uchenna R Ofoma, Edward K Chapnick

**Affiliations:** 1 Division of General Internal Medicine, Penn State Milton S. Hershey Medical Center, 500 University Drive, Hershey, PA 17033, USA; 2 Department of Infectious Diseases, Maimonides Medical Center, 4802 10th Avenue, Brooklyn, NY 11219, USA

## Abstract

Drug induced toxic epidermal necrolysis and Stevens Johnson syndrome are more commonly associated with medications such as sulfonamides, penicillin, anticonvulsants, oxicam non-steroidal anti-inflammatory drugs, allopurinol and corticosteroids. Isolated instances secondary to drugs outside of the aforementioned classes have also been reported. We report a case of probable toxic epidermal necrolysis induced by fluconazole in a 52 year old woman.

## Introduction

Adverse cutaneous reactions to drugs are common occurrences, affecting 2-3% of hospitalized patients [1]. Drug induced Toxic Epidermal Necrolysis (TEN) and Stevens Johnson Syndrome (SJS) are more commonly associated with medications such as sulphonamides, penicillin and other antibiotics, anticonvulsants, oxicam NSAIDS, allopurinol and corticosteroids. Isolated instances of TEN and SJS secondary to drugs that do not belong to the aforementioned classes have also been reported. A Medline literature search revealed only three previously reported cases of mucocutaneous drug reactions secondary to fluconazole therapy. We report a case of fluconazole induced toxic epidermal necrolysis.

## Case presentation

**Figure 1:**
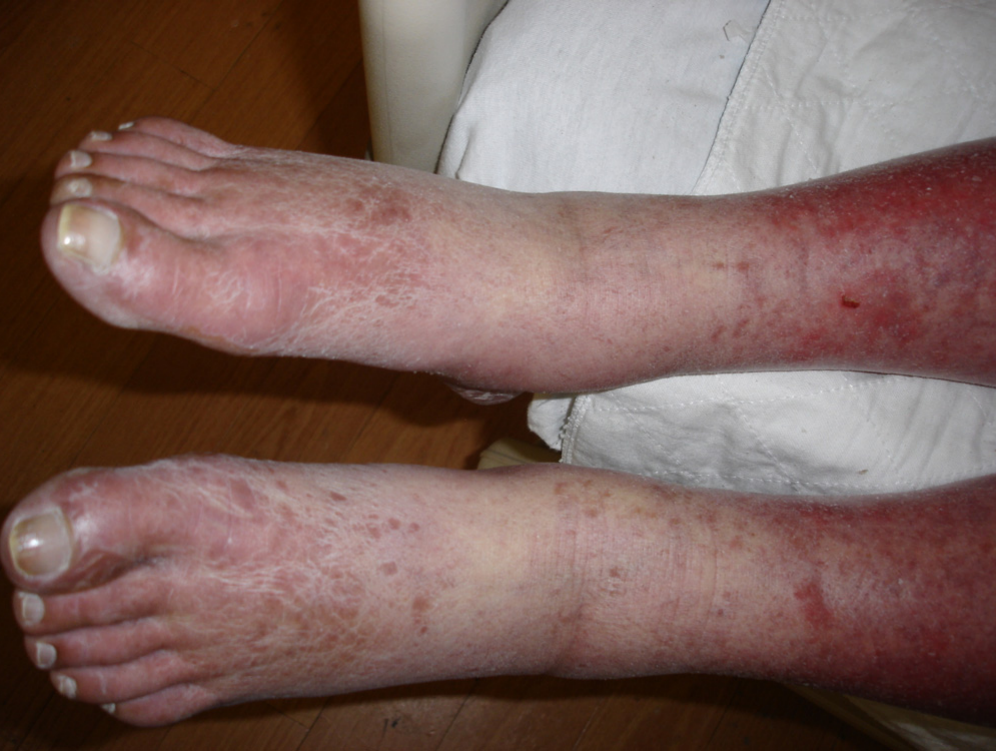
**TEN; Note the widespread dusky rash**.

**Figure 2:**
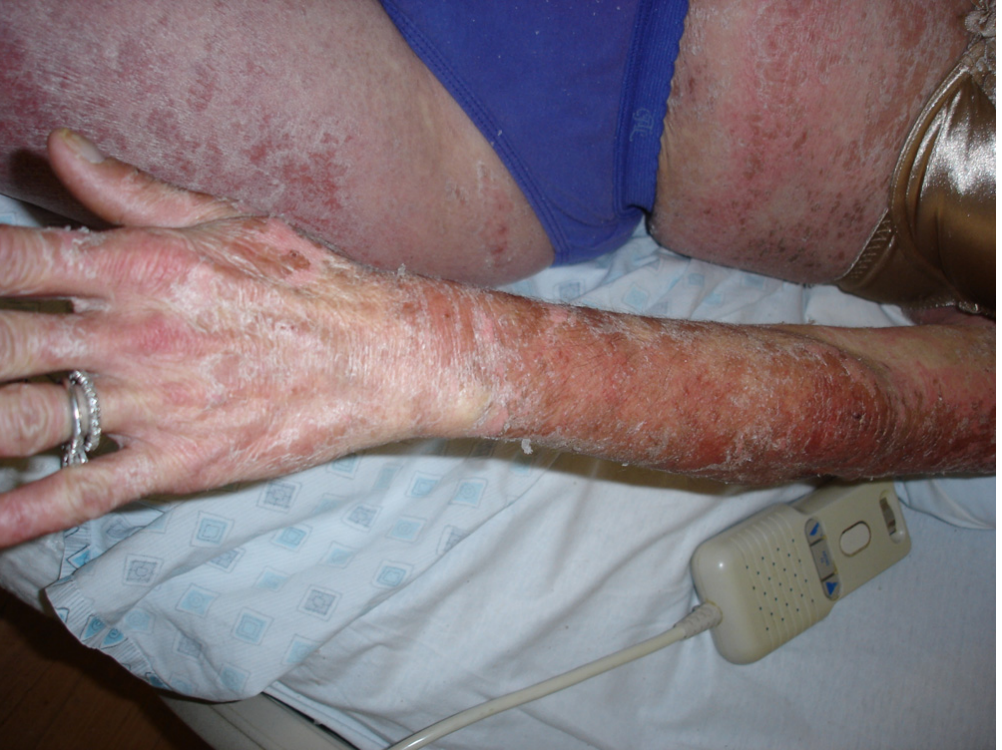
**TEN; Widespread rash with skin peeling off in left upper extremity, anterior trunk and lateral thigh**.

**Figure 3:**
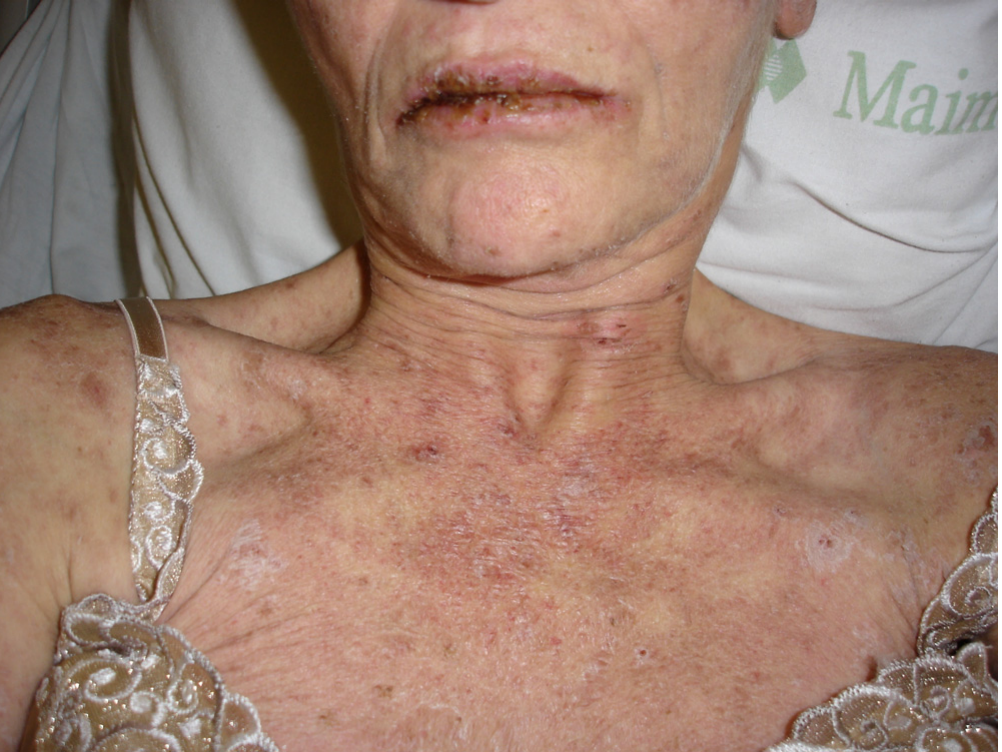
**TEN; Rash on anterior chest**. Note involvement of the mouth.

**Figure 4:**
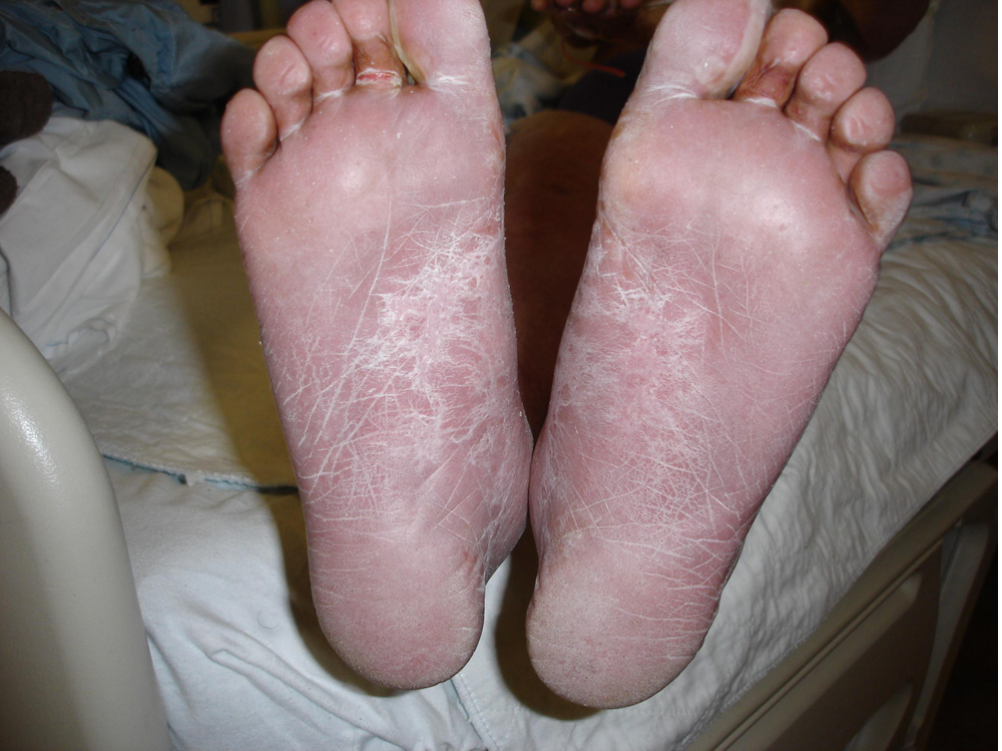
**TEN; More subtle rashes on plantar aspect of feet**. Note blistering of the skin overlying the metatarsophalangeal joints.

**Figure 5:**
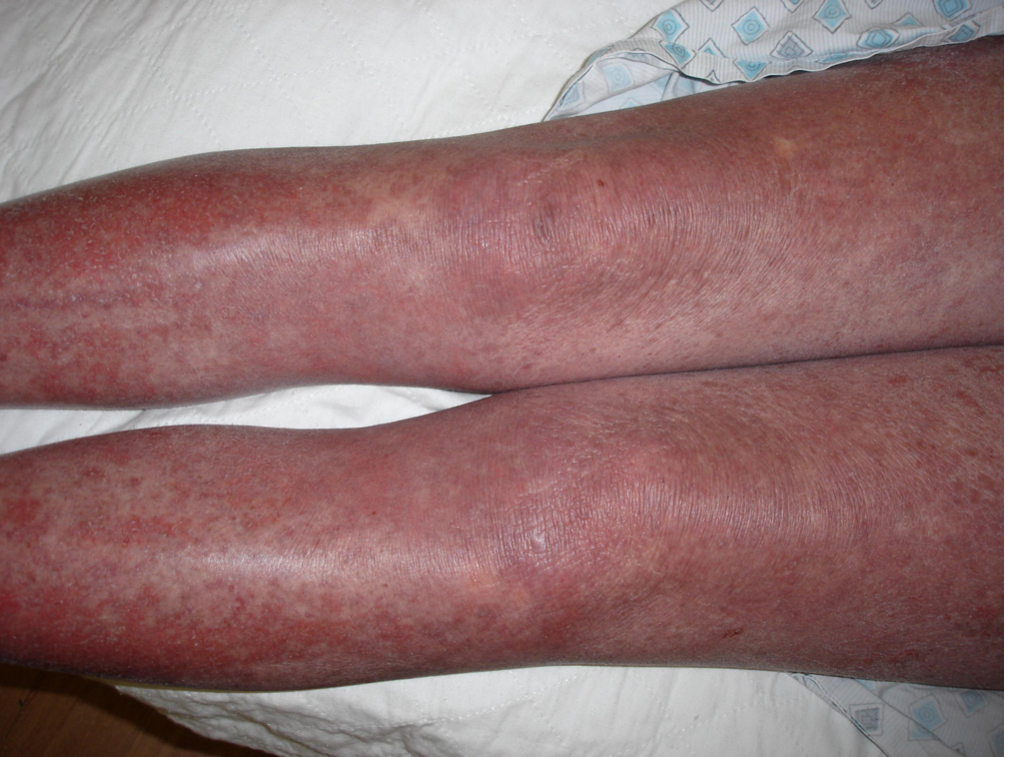
**TEN; Widespread rash involving the lower extremities**.

A 52-year-old unemployed Caucasian woman with a 45 pack year smoking history and past medical history significant for depression, peptic ulcer disease and herpes zoster presented with painful generalized body rash. The rash involved greater than 80% of the body surface area and occurred five days after beginning treatment with 150 mg daily dose of oral fluconazole for esophageal candidiasis. Medications prior to admission included sertraline and esomeprazole, which she had taken for at least one year without any recent change in dose. There were no prodromal symptoms and the patient was afebrile. Initially involving the anterior trunk, the rash later spread peripherally to involve the rest of the trunk, upper and lower extremities, face, palms and soles. She also developed marked lip and oral blisters. Nikolsky's sign was absent. Over a course of 6 days the rash progressed from ill defined discrete and confluent macules to blisters. (Figures 1, 2, 3, 4 and 5)

A diagnosis of fluconazole induced toxic epidermal necrolysis (Naranjo score 6) was made based on the clinical picture. A skin biopsy later revealed necrolysis of all epidermal layers with focal epidermal detachment. Laboratory studies revealed a normal WBC count (6.2), mildly elevated liver enzymes (ALT 45 IU/L, AST 61 IU/L ALK 135 IU/L) and pancreatic amylase (112 IU/L). Blood cultures grew no organisms. She declined HIV testing. In-hospital management included pain control, skin, mouth and eye care, intravenous hydration and withdrawal of fluconazole following which the lesions gradually resolved.

## Discussion

A response to a drug, which is noxious and unintended and which occurs at doses normally used for prophylaxis, diagnosis or therapy of disease, or for the modification of physiologic function, is classified as an adverse drug reaction [2]. Type A reactions are dose dependent and predictable whereas type B reactions (idiosyncratic) are dose independent. When such a response leads to changes in the structure and/or function of the skin, its appendages or mucous membrane, resulting in outcomes such as death, life-threatening events, hospitalization, disability or interventions to prevent permanent impairment or damage, a severe cutaneous adverse drug reaction is said to have occurred [3]. Classic severe drug reaction patterns include angioedema/anaphylaxis, exfoliative dermatitis, Stevens-Johnson syndrome and toxic epidermal necrolysis and drug hypersensitivity syndromes [4].

TEN and SJS are acute and life threatening disorders of unclear pathophysiology. Epidermal necrosis causes erosions of the mucous membranes, extensive detachment of the epidermis, and severe constitutional symptoms [1,5] Cases with less than 10% of the epidermis involved are designated as SJS; those with 30% involvement of the epidermis are labelled as TEN; and cases with between 10 and 30% involvement are defined as overlap SJS-TEN [6].

While SJS can be caused by medications and/or infection, TEN is almost always drug induced [1,5,7]. More than 100 drugs have been implicated as causes of SJS and TEN in case reports, but there is a very strong association with specific medications (Table 1) [4], in nearly 80% of cases [1,5,8-12] Sulfonamides, anticonvulsants and allopurinol are the most consistently associated.

**Table 1:** Drugs Associated with Stevens Johnson Syndrome and TEN

More Frequently	Less Frequently
Sulphadoxine	Cephalosporins

Sulphadiazine	Fluoroquinolones

Sulphasalazine	Vancomycin

Cotrimoxazole	Rifampin

Hydantoins	Ethambutol

Carbamazepine	Fenbufen

Barbiturates	Tenoxicam

Phenylbutazone	Tiaprofenic acid

Piroxicam	Diclofenac

Chlomezone	Sulindac

Allopurinol	Ibuprofen

Amithiozone	Ketoprofen

Aminopenicillins	Naproxen

Adapted from Nirken, MH. Stevens-Johnson syndrome and toxic epidermal necrolysis in adults. In: UpToDate, Rose, BD (Ed), UpToDate, Waltham, MA, 2006 [4]

The pathophysiologic mechanisms for SJS and TEN are still obscure but immunologic mechanisms, reactive drug metabolites and interactions between the two have been proposed [4]. Studies have suggested that the metabolic pathway of TEN involves an imbalance in activation and detoxification mechanisms. This condition may result from an inherited or acquired deficiency in phase 2 detoxification enzymes and from an increase in the cytochrome P450 (CP450) isoform(s) responsible for processing the culprit drug to reactive metabolites These metabolic idiosyncrasies can lead to increased levels of compounds serving as potential immunogens or showing a direct cytotoxic effect [13].

Underlying diseases, particularly those that compromise immunity, may also play a role in inducing SJS or TEN [14,15]. Patients with SLE or HIV have an increased incidence of these syndromes [15-17]. The higher incidence of TEN found in patients with AIDS may be due to glutathione deficiency, as glutathione conjugation to reactive metabolites is a major pathway of detoxification [13].

Fluconazole belongs to the azole group of antifungals, which act by reducing ergosterol synthesis by inhibiting fungal cytochrome P450 enzymes. It is utilized for most *Candida *infections, coccidioidal meningitis, treatment and secondary prophylaxis of cryptococcal meningitis and empiric treatment of critically ill patients. In addition, fluconazole is used prophylactically to reduce fungal disease in bone marrow transplant recipients. Fluconazole is also a known inhibitor of the human cytochrome P450 system particularly the isozymes CYP1A2 (weak), 2C9 (strong), 2C19 (strong) and 3A4 (moderate). We can therefore hypothesize that deficiency of phase 2 substrate (glutathione), as well as immunological response to antigens are the predominant mechanisms for this adverse drug reaction.

Fluconazole has previously been implicated twice in the medical literature as a cause of SJS or TEN. Of the two previously reported cases, one involved a 30-year-old homosexual man who developed SJS following treatment with fluconazole for oral candidiasis. He was found to be HIV-positive [18]. The other involved a 33-year-old HIV infected man who developed TEN following treatment with this agent for dysphagia due to recurrent oral thrush [19]. Lester et al [20], reported diffuse exfoliative drug reaction in a 30-year-old patient with the acquired immuno-deficiency syndrome (AIDS) who had been on fluconazole for oral thrush. This patient demonstrated mucosal sparing and a clear clinical distinction between widespread bullous fixed drug eruption and TEN could not be made. We report a case of severe, latent, type B TEN. Our patient had history suspicious for immunosuppression, probably secondary to HIV infection but declined testing. To our knowledge, this is the third reported case of SJS/TEN secondary to fluconazole.

## Abbreviations

ALK: alkaline phosphatase; ALT: alanine transaminase; AST: aspartate transaminase; NSAID: non-steroidal anti-inflammatory drugs; WBC: white blood cell.

## Competing interests

The authors declare that they have no competing interests.

## Authors' contributions

UO gathered the relevant data and wrote the primary and revised manuscripts. EC reviewed the manuscript for relevance, consistency and accuracy and made necessary corrections.

## Consent

Written informed consent was obtained from the patient for publication of this case report and accompanying images and is maintained in the patients medical records. A copy of the written consent is available for review by the Editor-in-Chief of this journal.
